# Methylation Status of the *Nanog* Promoter Determines the Switch between Cancer Cells and Cancer Stem Cells

**DOI:** 10.1002/advs.201903035

**Published:** 2020-01-23

**Authors:** Shupeng Liu, Kai Cheng, Hui Zhang, Ruijiao Kong, Shuo Wang, Chuanbin Mao, Shanrong Liu

**Affiliations:** ^1^ Department of Obstetrics and Gynecology Shanghai Tenth People's Hospital Tongji University Shanghai 200072 China; ^2^ Department of Laboratory Diagnostics Changhai Hospital Second Military Medical University Shanghai 200433 China; ^3^ Department of Laboratory Diagnostics Shanghai Fourth People's Hospital Affiliated to Tongji University School of Medicine Shanghai 200081 China; ^4^ Department of Chemistry and Biochemistry Stephenson Life Sciences Research Center University of Oklahoma 101 Stephenson Parkway Norman OK 73019‐5300 USA

**Keywords:** cancer stem cells, methylation, miRNA, Nanog, plasticity

## Abstract

Cancer stem cells (CSCs) are the main cause of tumor development, metastasis, and relapse. CSCs are thus considered promising targets for cancer therapy. However, it is hard to eradicate CSCs due to their inherent plasticity and heterogeneity, and the underlying mechanism of the switch between non‐CSCs and CSCs remains unclear. Here, it is shown that miR‐135a combined with SMYD4 activates Nanog expression and induces the switch of non‐CSCs into CSCs. The miR‐135a level, once elevated, lowers the methylation level of the CG5 site in the *Nanog* promoter by directly targeting DNMT1. SMYD4 binds to the unmethylated *Nanog* promoter to activate Nanog expression in Nanog‐negative tumor cells. The in vivo regulation of miR‐135a levels could significantly affect both the CSCs proportion and tumor progression. These findings indicate that DNA methylation of the *Nanog* promoter modulates the switch of non‐CSCs into CSCs under the control of the miRNA‐135 level. In addition, the related pathways, miR‐135a/DNMT1 and SMYD4, involved in these processes are potential targets for CSC‐targeted therapy.

## Graphical abstract

The conversion of non‐cancer stem cells (CSCs) to CSCs in tumors is found to follow the miR‐135a/DNMT1/Nanog signaling pathway and involve SMYD4. This discovery shows how DNA methylation mediates the plasticity of CSCs and why Nanog presents heterogeneous expression in tumor tissues. It suggests that the related pathway can be a target for CSC‐targeted cancer therapy.

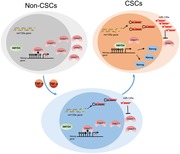

## 1 Introduction

Currently, cancer stem cells (CSCs), a subset of tumor cells with the capability of tumor initiation and self‐renewal, have been found in both hematopoietic and solid tumors.^1^, ^2^ Due to their ability to survive after therapy and thus fail the cancer therapy, CSCs were considered the seeds for tumor incidence, metastasis and relapse,^3^, ^4^ rendering them promising targets for cancer therapy.^5^

However, increasing evidence has shown that CSCs are phenotypically heterogeneous and thus are difficult to target, although CSCs in tumors uniformly express the stem cell transcription factor Nanog, the common molecular signature of tumor stem cells.^6^, ^7^, ^8^ First, CSCs in tumors from different human tissues show different surface markers. For example, acute myeloid leukemia CSCs are CD34^+^CD38^−^,^2^ brain tumor CSCs are CD133^+^ or CD15^+^,^9^, ^10^ and breast tumor CSCs are CD44^+^CD24^−^.^11^ Second, even CSCs from the same type of tumor also have different surface markers. For example, in liver cancer, tumor cells expressing ICAM‐1, CD133, CD24, and EpCAM were reported to be CSCs,^12^ and in breast cancer, the ALDH^hi^ and CD44^hi^CD24^lo^ CSC subsets both displayed CSC properties.^13^ However, the CSCs with different surface markers from the same type of tumors displayed distinct functional phenotypes. In liver cancer, CD24^+^ CSCs showed a higher tumorigenic ability than CD133^+^ and EpCAM^+^ CSCs.^14^ In breast cancer, ALDH^+^ CSCs were more quiescent, similar to luminal‐type normal breast stem cells, while CD44^+^CD24^−^ CSCs were more mesenchymal‐like, resembling basal‐type breast stem cells.^13^ In ovarian cancer, ALDH^+^CD133^+^ CSCs showed a higher tumor initiation ability than ALDH^+^CD133^−^ CSCs, although both subsets could induce tumor formation in mice.^15^ Based on these findings, we, along with others, hypothesized that CSCs in tumor tissues may be a subset of stem cells undergoing constant, dynamic changes, indicating their plasticity.^4^, ^16^ The phenotypic plasticity displayed on CSCs was considered the driving force for CSCs to contribute to tumor initiation, progression, and therapy resistance.^17^ Moreover, the inherently plastic characteristics make it difficult to eliminate CSCs via their surface markers or the heterogeneous functional phenotype used in tumor therapy. However, the mechanism underlying the CSC plasticity remains unclear.

It is well known that epigenetic regulation is involved in tumorigenesis. The methylome analysis revealed that the large hypermethylated regions in the normal cells become hypomethylated in the tumors.^18^ Tumor cells in nearly every type of cancer show global hypomethylation accompanied by hypermethylation at the distinct loci.^19^ DNA methylation modifiers, such as DNA methyltransferases (DNMTs) and ten‐eleven translocation (TET) proteins, can contribute to the aberrant methylation patterns in the tumor cells.^20^ Further studies reported that methylation of the CpG‐rich DNA region within the H1.0 gene silenced its expression and then promoted CSC maintenance.^21^ In addition, DNA methylation was also involved in establishing phenotypic and functional diversity in the tumor tissues. It was found that heterogeneous DNA methylation patterns were correlated with intratumor heterogeneity in several types of tumors, such as gliomas and esophageal squamous cell carcinomas.^22^ In chronic lymphocytic leukemia, high intratumor variability of DNA methylation patterns was reported to contribute to the heterogeneity within tumors.^23^ Furthermore, dysregulation of DNA methylation could induce CSC formation and sustain their phenotypes during tumor development and progression.^24^ For example, DNMT1 induced CSC formation and tumor cell growth in breast cancer by downregulating ISL LIM homeobox 1 (ISL1) expression.^25^ However, no correlation was observed between DNA methylation and CSC heterogeneity.

In the present study, we found that miR‐135a combined with SMYD4 contributed to the conversion between CSCs and non‐CSCs by activating Nanog expression through modulating its promoter methylation. Elevated expression of miR‐135a reduced *Nanog* promoter methylation by directly targeting DNMT1. SMYD4 bound to the unmethylated *Nanog* promoter to activate Nanog expression in Nanog‐negative tumor cells. In addition, targeting miR‐135a inhibited the CSC capacity of tumor cells both in vitro and in vivo. These findings uncovered how DNA methylation regulated the plasticity of CSCs and explained why Nanog showed heterogeneous expression in tumor tissues. They also indicated that the inflammatory microenvironment is involved in the epigenetic regulation of CSC plasticity and that the related pathways can be targets for CSC‐targeted therapy.

## 2 Results

### 2.1 CG5 Methylation of the *Nanog* Promoter Controlled By DNMT1 Determined Nanog Expression and Distinguished CSCs from Non‐CSCs in Tumors

**Figure 1:**
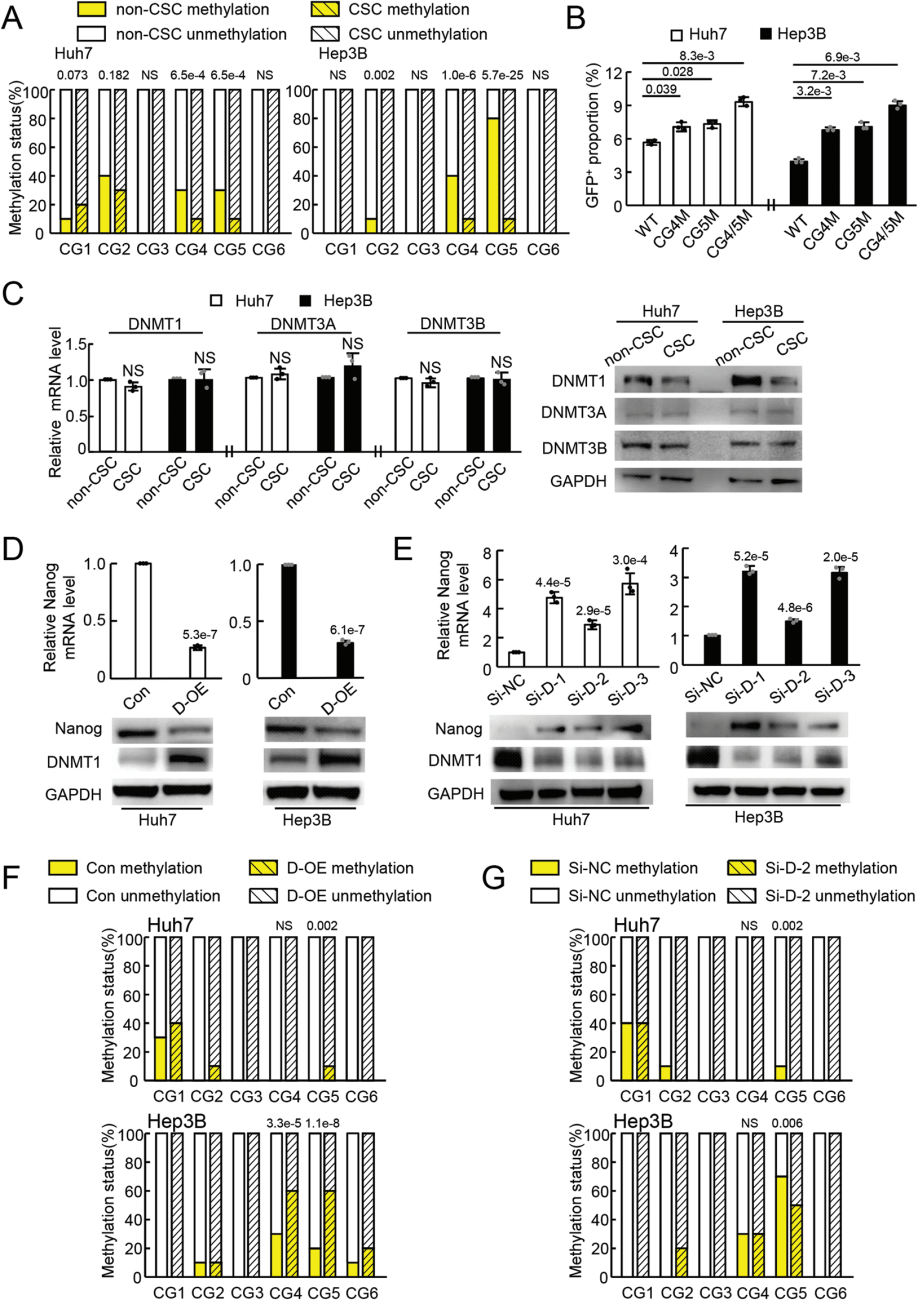
DNMT1 suppressed Nanog expression by methylating the *Nanog* promoter in tumor cells. A) BSP analysis showing different methylation patterns of the *Nanog* promoter between CSCs and non‐CSCs. Ten clones were sequenced for each CG in the *Nanog* promoter. B) FACS analysis of the GFP^+^ cell population in tumor cells transfected with plasmids harboring a *Nanog* promoter with or without a single nucleotide mutation at CG4 or/and CG5. C) DNMT (DNMT1, DNMT3A, and DNMT3B) expression in CSCs and non‐CSCs analyzed by qRT‐PCR and WB in triplicate. D,E) qRT‐PCR and WB analysis of Nanog expression in tumor cells with D) DNMT1 upregulation and E) DNMT1 downregulation in triplicate. F,G) Methylation pattern of the *Nanog* promoter in tumor cells with F) DNMT1 upregulation and G) DNMT1 downregulation assessed by BSP analysis. Ten clones were sequenced for each CG in the *Nanog* promoter. B–E) Representative data of triplicate experiments are shown as the mean ± standard deviation (SD). A,F,G) *P* value was assessed by Fisher's exact test. C–E) One‐way ANOVA with Dunnett‐*t* test in comparison with WT, or Student's *t* test. NS, no significant difference. Representative images of triplicate WB experiments are shown.

To exclude the confusion in distinguishing CSCs from non‐CSCs led by different surface markers in cancer cells, Nanog was thus taken as a molecular signature for us to identify CSCs.^6^, ^7^, ^8^ To conveniently identify CSCs from non‐CSCs according to Nanog expression, we conducted a plasmid, pH‐*Nanog* promoter‐GFP (pH‐NP‐GFP), which expressed GFP under the control of the *Nanog* promoter (hg38 chr12:7788192‐7789480). A minor population of tumor cells expressing GFP after pH‐NP‐GFP transfection was successfully observed (Figure S1A, Supporting Information), which was sorted to be ≈5–7% (GFP^+^, Figure S1B; upper panel, Supporting Information). Nanog expression was markedly higher in these fluorescence‐activated cell sorter (FACS)‐sorted GFP^+^ cells (GFP^+^) than in the GFP^−^ cells (GFP^−^) (Figure S1B; lower panel, Supporting Information). GFP^+^ and GFP^−^ cells sorted by FACS were identified as CSCs and non‐CSCs, respectively, in the following study. Bisulfate‐sequencing polymerase chain reaction (PCR) (BSP) was performed to investigate the methylation of CG dinucleotide (CG) in the human *Nanog* promoter region (hg38 chr12:7788192‐7789480) in CSC and non‐CSC subsets from both Huh7 and Hep3B cells. Among the 17 CGs in this region, two CG (CG4, CG5) sites showed a much lower methylation level in CSCs than in non‐CSCs, whereas the other sites showed either similar methylation levels or different methylation tendencies between the two subsets from both cell lines (**Figure** **1**A; Figure S1C, Supporting Information).

To further investigate whether the CGs (CG4, CG5) were involved in *Nanog* transcription, plasmids containing mutant CG4 and/or CG5 in the *Nanog* promoter were constructed using a pH‐NP‐GFP plasmid (WT). The G in both CG4 and CG5 was replaced by T in the plasmids to avoid methylation modulation at these sites (termed pH‐NP‐GFP‐CG4M, CG4M; pH‐NP‐GFP‐CG5M, CG5M; pH‐NP‐GFP‐CG4/5M, CG4/5M) (Figure S1D, Supporting Information). These plasmids were transfected into tumor cells, followed by assaying GFP expression using FACS. Compared with the pH‐NP‐GFP (WT)‐transfected tumor cells, the GFP^+^ cells had an increased rate of tumor cells transfected with plasmids harboring the G > T mutant at CG4 (CG4M), at CG5 (CG5M) and at both CG4 and CG5 (CG4/5M) (Figure 1B; Figure S1E, Supporting Information). These results indicate that mutations at CG4 and CG5 demethylated the promoter and that the methylation of CG4 and CG5 may be involved in *Nanog* expression in tumor cells.

Then, the expression of DNMTs (DNMT1, DNMT3A, and DNMT3B), known to be responsive to DNA methylation,^20^ was assessed in CSCs and non‐CSCs. Very surprisingly, no differences among all three DNMT mRNAs were observed between CSCs and non‐CSCs from both Huh7 and Hep3B cells (Figure 1C; left panel). However, western blot (WB) analysis displayed that the DNMT1 protein was significantly lower in CSCs than in non‐CSCs. However, no differences in the DNMT3A and DNMT3B proteins between these two subsets were observed (Figure 1C; right panel). These findings suggest that DNMT1, instead of DNMT3A and DNMT3B, modulates *Nanog* promoter methylation and modulates its expression in this scenario. For further verification, gain‐of‐function and loss‐of‐function assays were performed to modulate DNMT1 expression in tumor cells, and then Nanog expression was evaluated. Compared with that induced by pcDNA3.1 plasmid transfection (Con), DNMT1 upregulation induced by pcDNA‐H‐DNMT1 plasmid transfection (D‐OE) significantly reduced Nanog expression at the level of both mRNA and protein (Figure 1D; Figure S2A, Supporting Information). Compared with the Si‐negative control (NC) group (Si‐NC), siRNA transfection (Si‐D) significantly reduced DNMT1 and markedly increased Nanog expression at the mRNA and protein level (Figure 1E; Figure S2B, Supporting Information). Moreover, the CG methylation in the *Nanog* promoter was also investigated when DNMT1 expression was modulated in tumor cells. BSP analysis identified a higher methylation level of CG5 in both Huh7 and Hep3B tumor cells with DNMT1 overexpression than that in the respective control cells. However, an increase in CG4 methylation was found in only Hep3B tumor cells (Figure 1F; Figure S2C, Supporting Information). When DNMT1 was downregulated in tumor cells, only the methylation level of CG5 was decreased, but the methylation statues of other CGs, including CG4, were not changed (Figure 1G; Figure S2D, Supporting Information), suggesting the involvement of CG5 methylation in the regulation of Nanog expression. Moreover, whether CG5 methylation regulated by DNMT1 affects the transcriptional activity of the *Nanog* promoter was also investigated. The plasmids containing either the wild‐type or mutant *Nanog* promoter were cotransfected into tumor cells with pcDNA‐H‐DNMT1 or pcDNA3.1 to determine whether GFP expression was affected. FACS analysis showed that the number of GFP^+^ cells was reduced when pH‐NP‐GFP‐transfected tumor cells were further transfected with pcDNA‐H‐DNMT1 rather than with pcDNA3.1 (Figure S2E, Supporting Information). This reduction in the number of GFP^+^ cells induced by pcDNA‐H‐DNMT1 transfection was also observed in pH‐NP‐GFP‐CG4M‐transfected tumor cells (Figure S2E, Supporting Information). In pH‐NP‐GFP‐CG5M‐transfected tumor cells, no change was observed in the number of GFP^+^ cells when they were transfected with pcDNA‐H‐DNMT1 or pcDNA3.1 (Figure S2E, Supporting Information). This result indicates that CG5 methylation regulated by DNMT1 affected the transcriptional activity of the *Nanog* promoter. These data showed that the methylation status of the *Nanog* promoter regulated by DNMT1 controlled Nanog expression in tumor cells, enabling the identification of CSCs from non‐CSCs.

### 2.2 miR‐135a Negatively Regulates DNMT1 Expression to Reduce the Methylation Level of CG5 in the *Nanog* Promoter, Enhancing the CSC Capability of Tumor Cells

**Figure 2:**
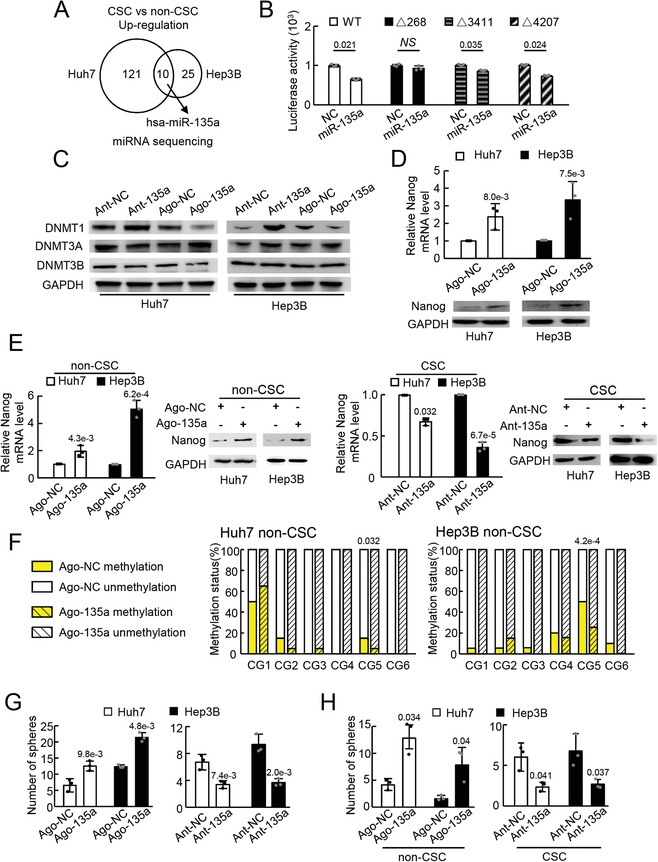
miR‐135a activated Nanog expression and enhanced the CSC capacity of tumor cells by targeting DNMT1. A) Schematic of upregulated miRNAs in CSCs compared with non‐CSCs screened by miRNA sequencing. Two biological replicates for each group were sequenced. Gene expression with a fold change >2 and false discovery rate < 0.05 was identified as significantly different. B) Binding of miR‐135a to DNMT1 mRNA sequence assessed by a dual‐luciferase assay in triplicate. C) DNMT protein expression in tumor cells with miR‐135a upregulation (Ago‐135a) or miR‐135a downregulation (Ant‐135a) analyzed by WB in triplicate. D) Nanog expression in tumor cells with miR‐135a upregulation (Ago‐135a) or the control (Ago‐NC) analyzed via qRT‐PCR and WB in triplicate. E) qRT‐PCR and WB analysis of Nanog expression in non‐CSCs with miR‐135a upregulation (Ago‐135a) and in CSCs with miR‐135a downregulation (Ant‐135a). F) Methylation pattern of the *Nanog* promoter in non‐CSCs with miR‐135a upregulation (Ago‐135a) or the control (Ago‐NC) assessed by BSP analysis. Twenty clones were sequenced for each CG in the *Nanog* promoter. G) Counts of spheres derived from tumor cells with miR‐135a upregulation (Ago‐135a) and miR‐135a downregulation (Ant‐135a) analyzed via sphere formation assay. H) Counts of spheres derived from non‐CSCs with miR‐135a upregulation (Ago‐135a) and CSCs with miR‐135a downregulation (Ant‐135a) analyzed via sphere formation assay. B–E) Representative data of triplicate experiments or of G,H) five fields in one of three experiments are shown as the mean ± SD. B,D,E,G,H) *P* value was assessed by Student's *t* test or F) Fisher's exact test. NS, no significant difference. Representative images of triplicate WB experiments are shown.

The inconsistent mRNA and protein expression level of DNMT1 in CSCs (Figure 1C) suggested the involvement of posttranscriptional mechanisms such as participation of microRNAs (miRNAs) in DNMT1 regulation. Differentially expressed miRNAs between CSCs and non‐CSCs were thus screened via RNA‐seq analysis. Ten miRNAs were identified to be upregulated in CSCs from both Huh7 and Hep3B cells, and among them, miR‐135a was previously reported to promote tumor cell metastasis by our group (**Figure** **2**A; Figure S3A, Supporting Information).^26^ In addition, CSCs are known to contribute substantially to tumor metastasis, and miR‐135a was thus selected for further investigation. The following quantitative reverse‐transcription PCR (qRT‐PCR) analysis confirmed a higher level of miR‐135a expression in CSCs enriched by sphere formation assays and FACS sorting than in the corresponding control cells (Figure S3B, Supporting Information).

Then, we investigated whether miR‐135a regulated DNMT1 expression. The prediction tool RNA22 V2.0 was used to analyze whether miR‐135a binds to DNMT1 mRNA.^27^ Three potential miR‐135a binding sites were found in the DNMT1 coding sequence with the 92% sensitivity and 8% specificity settings (*P* > 0.05) (Figure S3C, Supporting Information). DNMT1 mRNA sequences with or without miR‐135a binding sites were subsequently cloned into pmiR‐GLO to generate luciferase reporter plasmids. The luciferase reporter plasmids were cotransfected into Huh7 cells with miR‐135a mimics and NC constructs. The luciferase reporter assays showed that miR‐135a overexpression suppressed the firefly luciferase activity of the reporter plasmid containing the wild‐type DNMT1 mRNA sequence (pmiR‐GLO‐WT) compared with the luciferase activity of the NC (Figure 2B). miR‐135a suppressed the firefly luciferase activity of the luciferase reporter plasmids devoid of site 3411 (pmiR‐GLO‐Δ3411) or site 4207 (pmiR‐GLO‐Δ4207), but miR‐135a did not reduce the firefly luciferase activity of the plasmid without site 268 (pmiR‐GLO‐Δ268) (Figure 2B). These results suggest that miR‐135a regulates DNMT1 by binding to its mRNA at site 268. For further verification, synthesized miR‐135a agomir (Ago‐135a) or antagomir (Ant‐135a) were transfected into tumor cells (Huh7 and Hep3B), and DNMT1 expression was assessed at both the mRNA and protein levels. The results showed that compared with the relative NC transfection, Ago‐135a transfection reduced DNMT1 protein expression, while Ant‐135a transfection increased DNMT1 protein expression (Figure 2C). In addition, no apparent changes in DNMT3A and DNMT3B were observed in tumor cells (Figure 2C). The mRNA levels of these three molecules displayed no significant change (Figure S3D, Supporting Information). Moreover, miR‐135a overexpression increased Nanog expression in tumor cells (Figure 2D).

Furthermore, the regulation of Nanog by miR‐135a was verified in non‐CSCs and CSCs. Ago‐135a and Ant‐135a were transfected into non‐CSCs and CSCs to up‐ and down‐ regulate Nanog expression, respectively (Figure 2E). Then, to investigate whether miR‐135a could modulate methylation of the *Nanog* promoter, non‐CSCs with or without ectopic miR‐135a expression were analyzed by BSP. Lower CG5 methylation levels were observed in the *Nanog* promoter in non‐CSCs transfected with Ago‐135a from both Huh7 and Hep3B than in the corresponding control cells, while CG4 showed lower methylation levels in only Hep3B non‐CSCs with ectopic miR‐135a expression; other CGs showed different methylation statuses between non‐CSCs from Huh7 and Hep3B cells (Figure 2F; Figure S3E, Supporting Information).

Moreover, whether miR‐135a could impact CSC properties was also investigated due to its role in Nanog regulation. For this purpose, tumor cells transfected with either Ago‐135a or Ant‐135a were evaluated by sphere formation assay. Larger and more spheres were observed in cells transfected with Ago‐135a, and fewer spheres were observed in cells transfected with Ant‐135a (Figure 2G; Figure S4A,B, Supporting Information). Non‐CSCs transfected with Ago‐135a and CSCs transfected with Ant‐135a were also analyzed by sphere formation assay. More spheres were derived from non‐CSCs transfected with Ago‐135a than with the relative NC (Figure 2H; left panel and Figure S4C, Supporting Information). Immunofluorescence (IF) staining revealed that Nanog was expressed in spheres derived from non‐CSCs transfected with Ago‐135a (Figure S4D, Supporting Information), further verifying the induction of Nanog expression by miR‐135a. In addition, fewer spheres were derived from CSCs after transfection with Ant‐135a (Figure 2H; right panel and Figure S4E, Supporting Information). These results showed that miR‐135a upregulated Nanog expression and thus enhanced the CSC capability of tumor cells by reducing the methylation level of CG5 in the *Nanog* promoter through targeting DNMT1 in vitro.

### 2.3 Ectopic Expression of miR‐135a Modulates CSC Proportion, Influencing the Tumor Growth In Vivo

**Figure 3:**
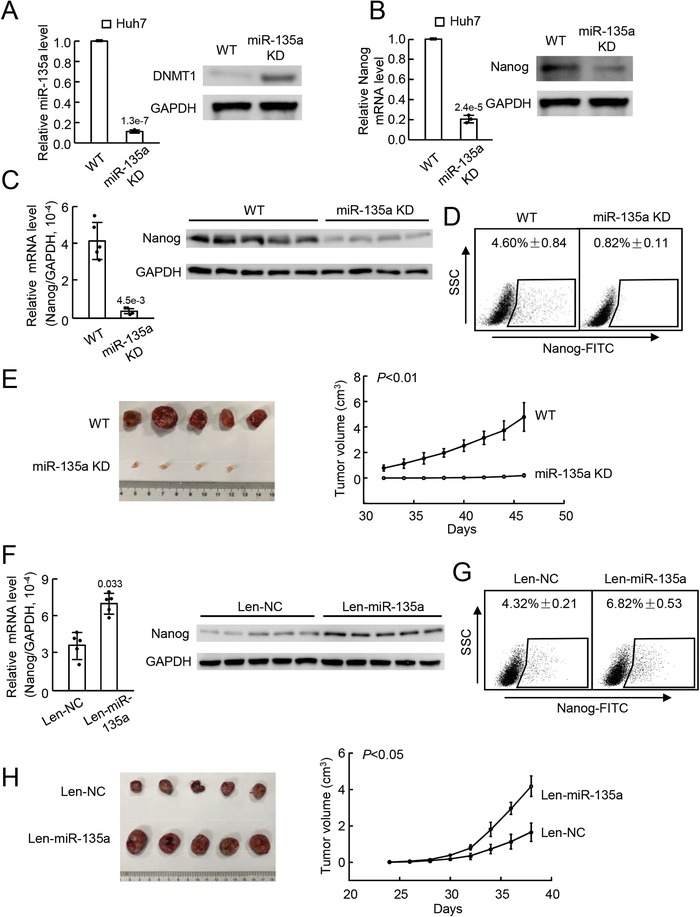
miR‐135a upregulated Nanog expression and enhanced CSC capacity of tumor cells in vivo. A) Expression of miR‐135a (left panel) and the DNMT1 protein (right panel) in Huh7 cells without (WT, wild‐type) or with miR‐135a knockdown (miR‐35a KD) assessed by qRT‐PCR or WB in triplicate. B) Expression of Nanog in Huh7 cells without (WT) or with miR‐135a knockdown (miR‐35a KD) assessed by qRT‐PCR and WB in triplicate. C) Expression of Nanog assessed by qRT‐PCR and WB and D) Nanog^+^ cell rate assessed by FACS analysis in tumors derived from tumor cells without (WT) or with miR‐135a knockdown (miR‐135a KD). *n* = 5 in WT, *n* = 4 in miR‐135a KD. E) Representative image (left panel) and tumor growth curve (right panel) of tumors derived from tumor cells with (miR‐135a KD) or without (WT) miR‐135a knockdown in mice, *n* = 5 each group. F) Expression of Nanog assessed by qRT‐PCR and WB and G) the Nanog^+^ cell rate was assessed by FACS analysis in tumors with (Len‐miR‐135a) or without (Len‐NC) miR‐135a upregulation, *n* = 5 each group. H) Representative image (left panel) and growth curve (right panel) of tumors with with (Len‐miR‐135a) or without (Len‐NC) miR‐135a upregulation in mice, *n* = 5 each group. The image of GAPDH from WB in (A) was also used in (B). Representative data are shown as the mean ± SD. *P* value was assessed by Student's *t* test.

Now that we found that miR‐135a could promote the conversion of non‐CSCs into CSCs in vitro, we intended to verify the findings in vivo using a mouse model. To establish a mouse model with miR‐135a downregulation in vivo, Huh7 cell lines with miR‐135a knockdown were constructed using the CRISPR/Cas9 system. After validation of miR‐135a downregulation in knockdown cells (miR‐135a KD) (**Figure** **3**A; left panel), a significant upregulation of the DNMT1 protein (Figure 3A; right panel) and a downregulation of Nanog were observed in miR‐135a‐knockdown tumor cells (Figure 3B). Then, Huh7 cells without or with miR‐135a knockdown were subcutaneously injected into the lower back regions of mice to establish a mouse model. Tumors were obtained for further analysis after reaching 2 cm in diameter. Expression analysis showed lower expression of Nanog and miR‐135a in tumors derived from miR‐135a knockdown (miR‐135a KD) Huh7 cells than in those from wild‐type (WT) Huh7 cells (Figure 3C; Figure S4F, Supporting Information). FACS analysis displayed a lower rate of Nanog^+^ cells in miR‐135a KD tumors than in WT controls (Figure 3D), suggesting that CSC reduction was induced by miR‐135a downregulation. In addition, the miR‐135a KD tumors were much smaller and grew more slowly than did the WT controls, which further validated the reduction of CSCs in tumors with miR‐135a knockdown (Figure 3E; Figure S4G, Supporting Information). Additionally, an in vivo mouse model with miR‐135 upregulation was established. When tumors were formed, lentivirus expressing mature miR‐135a (Len‐miR‐135a) was injected in situ into the tumors to upregulate miR‐135a in vivo, and lentivirus expressing the NC sequence (Len‐NC) was used as a control. Tumors (up to 2 cm in diameter) were obtained from sacrificed mice for further analysis. miR‐135 overexpression in the tumors injected with len‐miR‐135a (Len‐miR‐135a) was observed (Figure S4H, Supporting Information), and Nanog expression was upregulated at both the mRNA and protein levels compared with the expression in the control tumors (Len‐NC, Figure 3F). FACS analysis showed that the rate of Nanog^+^ cells was higher in the len‐miR‐135a group than in the Len‐NC group (Figure 3G). In addition, tumors injected with len‐miR‐135a showed a larger volume and a greater growth capability than those injected with Len‐NC (Figure 3H; Figure S4I, Supporting Information), further verifying that more CSCs were induced by len‐miR‐135a administration.

Above all, these data indicated that miR‐135a expression increased Nanog expression in tumor cells and enhanced the CSC ability of tumor cells in vivo.

### 2.4 SMYD4 Transactivated Nanog Expression by Binding to the Unmethylated Site of the *Nanog* Promoter

**Figure 4:**
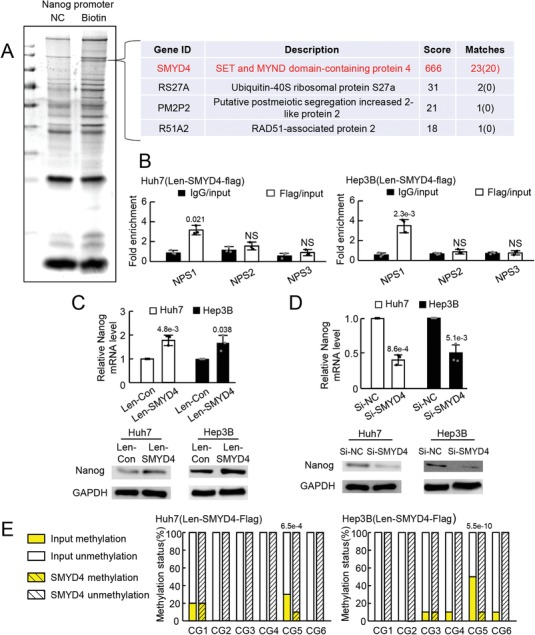
SMYD4 upregulated Nanog expression by binding to the unmethylated *Nanog* promoter. A) Proteins binding to the *Nanog* promoter identified by DNA pull down and mass spectrometry analyses. Representative images from triplicate experiments are shown. NC, negative control, no biotin labeled; Biotin, biotin labeled. B) ChIP analysis showing the binding of SMYD4 to the *Nanog* promoter. NPS1, *Nanog* promoter sequence including CG5; NPS2, and NPS3, *Nanog* promoter sequence out of NPS1. NS, no significant difference. Expression of Nanog in tumor cells with C) SMYD4 upregulation and D) downregulation assessed by qRT‐PCR and WB in triplicate. Representative data from triplicate experiments are shown as the mean ± SD. E) Methylation of CGs from sequences enriched by SMYD4 and input controls assessed by BSP analysis. Ten clones were sequenced for each CG in the *Nanog* promoter. *P* value was assessed by B–D) Student's *t* test or E) Fisher's exact test.

Among the 17 CGs in the *Nanog* promoter, CG4/5 was found to be differentially methylated between CSC and non‐CSC. As DNA methylation interferes with the DNA binding of transcription factors to regulate gene expression,^28^ we intended to identify transcription factors that transactivate Nanog expression by binding to its unmethylated promoter. A DNA pull‐down assay was thus performed using a *Nanog* promoter, which was used for methylation analysis. The specifically precipitated proteins by the *Nanog* promoter were subject to mass spectrometric analysis (**Figure** **4**A; left panel). Among the identified proteins, SET and MYND domain‐containing protein 4 (SMYD4) was selected for further verification because its confidence was the highest (Figure 4A; right panel). To consolidate the binding of SMYD4 to the *Nanog* promoter, tumor cells expressing SMYD4‐Flag fusion proteins via lentivirus infection (Figure S5A, Supporting Information) were sent for chromatin immunoprecipitation (ChIP) analysis using magnetic anti‐Flag‐beads. Three sets of primers were used in the following qRT‐PCR analysis; one set amplified the sequence including CG5 in the *Nanog* promoter (*Nanog* promoter sequence 1, NPS1) and the other two sets amplified the sequence out of NPS1 in the *Nanog* promoter (NPS2 and NPS3). Compared with the IgG control beads, the anti‐Flag beads enriched NPS1 but did not enrich the other two sequences (NPS2 and NPS3) (Figure 4B), suggesting the binding of SMYD4 to the *Nanog* promoter at the sequence including CG5.

To verify whether the binding of SMYD4 to the *Nanog* promoter transactivated Nanog expression, SMYD4 expression in tumor cells was modulated, and Nanog expression was assessed. The results showed that the upregulation of SMYD4 enhanced Nanog expression at both the mRNA and protein levels in tumor cells (Figure 4C). The downregulation of SMYD4 by siRNA transfection reduced the mRNA and protein expression of Nanog (Figure 4D; Figure S5B, Supporting Information). These results indicated that SMYD4 transactivated Nanog expression in tumor cells by binding to its promoter. To further evaluate the methylation level of the SMYD4 binding site in the *Nanog* promoter, *Nanog* promoters enriched by SMYD4 in the ChIP assay were analyzed by BSP. The methylation level of CpGs in the SMYD4‐enriched sequence was found to be much lower than that in the input controls (Figure 4E), suggesting a preference for SMYD4 to bind to unmethylated sequences. These data indicated that SMYD4 transactivated Nanog expression in tumor cells by binding to its unmethylated promoter site.

## 3 Discussion

In the present study, we found that non‐CSCs (Nanog^−^) could express the stem cell transcriptional factor Nanog and display the characteristics of CSCs. The miR‐135a/DNMT1 pathway was involved in the conversion of non‐CSCs into CSCs, and reduced CG5 methylation of the *Nanog* promoter; Nanog expression was transcribed by SMYD4 specifically bound to the unmethylated *Nanog* promoter CG5 site, indicating that CSCs arose de novo from non‐CSCs.

**Figure 5:**
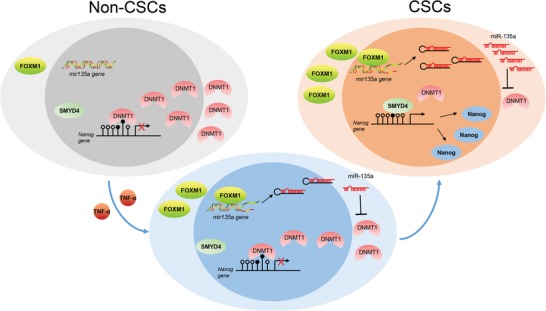
Schematic illustration of a mechanism by which DNA methylation of the *Nanog* promoter modulated the switch of non‐CSCs into CSCs under the control of the miRNA‐135 level. The miR‐135a level, once elevated, lowered the methylation level of the CG5 site in the *Nanog* promoter by directly targeting DNMT1. SMYD4 bound to the unmethylated *Nanog* promoter to activate Nanog expression in Nanog‐negative tumor cells. miR‐135a expression was upregulated by elevated FOXM1, the latter was activated by TNF‐α administration. The TNFα/FOXM1/miR‐135a/DNMT1 pathway was involved in the conversion of non‐CSCs into CSCs.

Our findings indicate that the methylation status of the CG5 site in the *Nanog* promoter in tumor cells distinguished CSCs from non‐CSCs and thus could serve as an indicator to confirm the conversion of non‐CSCs into CSCs. The presence of functionally distinct tumor cell subsets (tumorigenic CSCs and nontumorigenic non‐CSCs) has been the basic observation in many tumors over the decades. According to the plasticity model of CSC heterogeneity, the heterogeneity of CSCs resulted from the stochastic switch between distinct cell states.^29^ This model complies with the following two possibilities: CSCs arise de novo from non‐CSCs and CSCs adopt their phenotypes under certain given conditions. CSC switches between different functional phenotypes were well reported.^30^, ^31^ To date, very little is known about the conversion of non‐CSCs into CSCs due to the lack of appropriate markers to identify non‐CSCs and CSCs. Nanog is well known as the main stemness‐related transcription factor.^8^ Nanog is generally expressed in CSCs with different phenotypes, and thus, is now identified as the universal molecular feature of CSCs in different cancers.^32^, ^33^, ^34^ In our present study, the methylation status of the *Nanog* promoter CG5 site was identified as an indicator to mark the difference between CSCs and non‐CSCs. We further demonstrated that DNA methylation and miRNA, two main types of epigenetic regulation, were also involved in the conversion of non‐CSCs into CSCs. Repressed methylation of the *Nanog* promoter CG5 site by miR‐135a activated Nanog expression in non‐CSCs and transformed non‐CSCs into CSCs. We and another group previously found that FOXM1 activated miR‐135a transcription and TNF‐α upregulated FOXM1 expression in hepatocellular carcinoma (HCC).^26^, ^35^ As is well known, the inflammatory microenvironment has pivotal roles in tumor development and CSC maintenance.^36^ Among components of the inflammatory microenvironment, TNF‐α is one of the main proinflammatory cytokines that increases inflammation and promotes tumor cell growth.^37^ Under our experimental settings, we confirmed these findings and verified that TNF‐α enabled non‐CSCs (Nanog^−^) to transform into CSCs (Nanog^+^) by activating Nanog expression through elevating miR‐135a expression and depressing the methylation of the *Nanog* promoter (Data not shown). Our findings put forward a novel pattern (**Figure** **5**) by which DNA methylation of the *Nanog* promoter modulated the switch of non‐CSCs into CSCs under the control of the miRNA‐135 level.

Our studies promoted further questions in the CSCs field. For example, why are non‐CSCs in culture converted into CSCs via Nanog activation in vitro? In fact, CSCs, though as a small fraction of cell population, remain detectable both in conventional tumor cell cultures and in sorted non‐CSC cultures. Under our experimental conditions, we observed that some sorted Nanog^−^ cells cultured in vitro re‐expressed Nanog and were converted into Nanog^+^ cells even without any treatment (Figure 2E). Hence, more thorough investigation is needed to uncover the underlying mechanisms.

## 4 Conclusions

Collectively, we reported the conversion of non‐CSCs to CSCs in HCC and determined the pivotal role of the miR‐135a/DNMT1/Nanog signaling pathway in this conversion. The results also suggest epigenetic modifiers as rational therapeutic targets to inhibit tumor relapse and to improve the prognosis of patients.

## 5 Experimental Section

##### Cell Lines and Mice

The human liver cancer cell lines Huh7 and Hep3B were maintained in high‐glucose Dulbecco's Modified Eagle Medium (DMEM, Gibco) containing 10% fetal bovine serum (Gibco BRL), 100 mg mL^−1^ penicillin G, and 50 µg mL^−1^ streptomycin (Gibco BRL) in the incubator (5% CO_2_, 37 °C). The cell lines were authenticated using short tandem repeat analysis by Huake Gene Technology Co., LTD., Beijing. Male nude mice (4 weeks old) were purchased from the Transgenic Animal Research Center of the Second Military Medical University. All mice were maintained in a pathogen‐free facility and used in accordance with the institutional guidelines for animal care. The animal work was approved by the Institutional Animal Care and Use Committee of the Second Military Medical University.

##### Quantitative Reverse‐Transcription PCR

Total RNA was extracted from tumor cell lines or tissues using TRIzol Reagent according to the manufacturer's instructions (Invitrogen, Carlsbad, CA). Reverse transcription was performed using the PrimeScript 1st Strand cDNA Synthesis Kit (TaKaRa Bio, Inc.) according to the manufacturer's instructions. Reverse transcription of miRNAs was performed using a miRcute miRNA First‐strand cDNA Synthesis Kit (TIANGEN Biotech (Beijing) Co., Ltd.). The SYBR Premix Ex Taq (TaKaRa Bio, Inc.) was used for reverse‐transcription PCR (qRT‐PCR) with the LightCycler 480 II (Roche, USA). All primers used in qRT‐PCR are listed in Table S1 in the Supporting Information. The gene expression levels were calculated relative to the expression of GAPDH (for mRNA) or U6 (for miRNA) using the 2^−ΔΔ^ *^c^* ^t^ or 2^−Δ^ *^c^* ^t^ method.

##### Western Blot Analysis

The total proteins (100 µg) were extracted using protein lysates containing protease inhibitors and were resolved in 10% sodium dodecyl sulfate (SDS)‐poly‐acrylamide gels and transferred electrophoretically to polyvinylidene fluoride (PVDF) membranes. 5% skim milk was used to block the PVDF membranes and dilute the primary antibodies (Table S2, Supporting Information). The blots were then incubated with the corresponding secondary antibody and visualized by enhanced chemiluminescence using ImageQuant LAS4010 (GE Healthcare).

##### Transient Transfection

A Lipofectamine 3000 kit (Invitrogen, Carlsbad, CA) was used for transient transfection according to the manufacturer's instructions. Plasmid transfection was performed when the cell density reached 70–80% confluence in 6‐well plates. For cotransfection, different plasmids were mixed at a 1:1 ratio for a total amount of 4 µg and then incubated with Lipofectamine. The cells were harvested for subsequent analyses 24 h after transfection. For miRNA transfection, cells (2 × 10^5^) were grown to a density of 50–60% in 6‐well plates, transfected with 100 pmol miRNAs, and harvested for the following analyses 24 h after transfection.

##### Flow Cytometry Analysis and Single‐Cell Sorting

To analyze the percentage of tumor cells expressing Nanog in tumor tissues, a single‐cell suspension was obtained by digesting tumor tissues with 1% collagenase (Sigma‐Aldrich, C2139), stained with the anti‐Nanog antibody listed in Table S2 (Supporting Information), and then analyzed with the MoFlo XDP flow cytometer (Beckman Coulter, High Wycombe, UK).

To sort CSCs and non‐CSCs from tumor cells, the pH‐*Nanog* promoter‐GFP plasmids, which expressed GFP under the *Nanog* promoter, were transfected into tumor cells. Twenty‐four hours after transfection, tumor cells were harvested and sorted with the MoFlo XDP flow cytometer. Sorted CSCs and non‐CSCs were suspended in DMEM supplemented with 10% fetal bovine serum and sent for further culture or analysis.

##### RNA Sequencing Analysis

Analysis of miRNA expression in sorted CSCs and non‐CSCs, including total RNA extraction, RNA sequencing and bioinformatics data analysis, were performed by Shanghai Novelbio, Ltd.

##### ChIP Assay

A ChIP assay kit (Millipore Corporation, Billerica, MA, USA) was applied for ChIP assays following the manufacturer's protocol. Briefly, the cells were treated with formaldehyde to cross‐link the protein with the DNA to ensure coprecipitation. The lysate pellets were ruptured and sonically processed to shear the chromatin to a detectable size. The protein‐DNA complexes were immunoprecipitated with the appropriate antibodies (Table S2, Supporting Information). The immunoprecipitates were then dissolved in 20 µL of double‐distilled water for further PCR analysis. Standard qRT‐PCR amplifications were performed using specific primers (ChIP primers, Table S1, Supporting Information).

##### DNA Pull‐Down Assay

A DNA pull‐down assay was performed according to standard procedures. The *Nanog* promoter sequence was cloned by PCR (NP primers listed in Table S1, Supporting Information) and labeled with biotin during amplification using biotin‐labeled NP primers. The *Nanog* promoter sequence without biotin labeling was used as a NC in the following pull‐down assays. Protein was obtained from tumor cell lysates and was incubated with biotin‐labeled or unlabeled DNA sequences. Then, the DNA–protein complex was enriched by Nucleic‐Acid Compatible Streptavidin Magnetic Beads (Fisher Scientific, 20164Y), and the enriched proteins were sent for mass spectrometry analysis by Jingjie PTM Biolab (Hangzhou) Co., Ltd., China.

##### Sphere Formation Assay

Tumor cells (1 × 10^5^) were plated into 6‐well plates coated with polyHEMA (Sigma)‐ and cultured in DMEM/F12 medium (Gibco) supplemented with 20 ng mL^−1^ IGF (PeproTech, USA), 1.0 ng mL^−1^ bFGF (Gibco), and 20 ng mL^−1^ EGF (AbD Serotec) for one week. Spheres were then used in the subsequent experiments.

##### Athymic Nude Mouse Model

The tumor mouse models were established as previously described.^38^ In brief, tumor models were established by subcutaneously injecting tumor cells (1 × 10^6^ for each mouse) into the lower back regions of mice. For gain‐of‐function analysis of miR‐135a in vivo, 7 days after tumor implantation, mice were randomly assigned to 2 groups (*n* = 5, each group) and injected intratumorally with lentivirus expressing miR‐135a or NC once a week. For loss‐of‐function analysis of miR‐135a in vivo, Huh7 cells with or without miR‐135a knockdown were injected into mice. Tumors were measured twice a week after tumors were derived. The tumor growth curve was plotted using the tumor volume, which was calculated according to *V* = *L* × *W* × *H*. Mice were sacrificed when tumors in either group were up to 2 cm in diameter, and the tumors were obtained for further analysis.

##### Immunofluorescence Staining

Indirect immunofluorescence staining was performed to analyze Nanog expression in spheres using antibodies listed in Table S2 (Supporting Information) as previously described.^39^

##### Bisulfite Sequencing PCR

Bisulfite sequencing PCR (BSP) was performed to analyze the methylation of CGs in the *Nanog* promoter as previously reported.^40^ Briefly, DNA from sorted CSCs and non‐CSCs was treated with sodium bisulfite to convert unmethylated cytosine into uracil using an EpiTect Bisulfite Kit (Qiagen, Hilden, Germany). Converted DNA was sent for PCR using the BSP primers listed in Table S1 (Supporting Information). The PCR product was inserted into T pMD‐18‐T (TaKaRa) according to the manufacturer's instructions, sequenced using Sanger sequencing, and analyzed using BiQ_Analyzer software by OE Biotech, Inc., Shanghai, China.

##### Statistical Analysis

Statistical analyses were performed with SPSS 13.0 for Windows (SPSS). Statistical tests including Student's *t* test, one‐way ANOVA and Fisher's exact test were applied as indicated. *P* < 0.05 was considered statistically significant.

## Conflict of Interest

The authors declare no conflict of interest.

## Supporting information

Supporting Information
