# Supplementary material for: Methylation Status of the Nanog Promoter Determines the Switch between Cancer Cells and Cancer Stem Cells
Source: Adv Sci (Weinh). 2020 Jan 23;7(5):1903035. doi: 10.1002/advs.201903035 (PMC7055559; doi:10.1002/advs.201903035)
Supplement: Supplementary file 1 — Supporting Information [file ADVS-7-1903035-s001.pdf]

Copyright WILEY-VCH Verlag GmbH & Co. KGaA, 69469 Weinheim, Germany, 2020.

ADVANCED SCIENCE
Open Access

Supporting Information

for *Adv. Sci.,* DOI: 10.1002/advs.201903035

Methylation Status of the *Nanog* Promoter Determines the Switch between Cancer Cells and Cancer Stem Cells

Shupeng Liu, Kai Cheng, Hui Zhang, Ruijiao Kong, Shuo Wang, Chuanbin Mao, and Shanrong Liu**

Copyright WILEY-VCH Verlag GmbH & Co. KGaA, 69469 Weinheim, Germany, 2018.

Supporting Information

Methylation Status of the *Nanog* Promoter Determines the Switch between Cancer Cells and Cancer Stem Cells

Shupeng Liu, Kai Cheng, Hui Zhang, Ruijiao Kong, Shuo Wang, Chuanbin Mao, Shanrong Liu**

Supplementary Figures
Figure S1

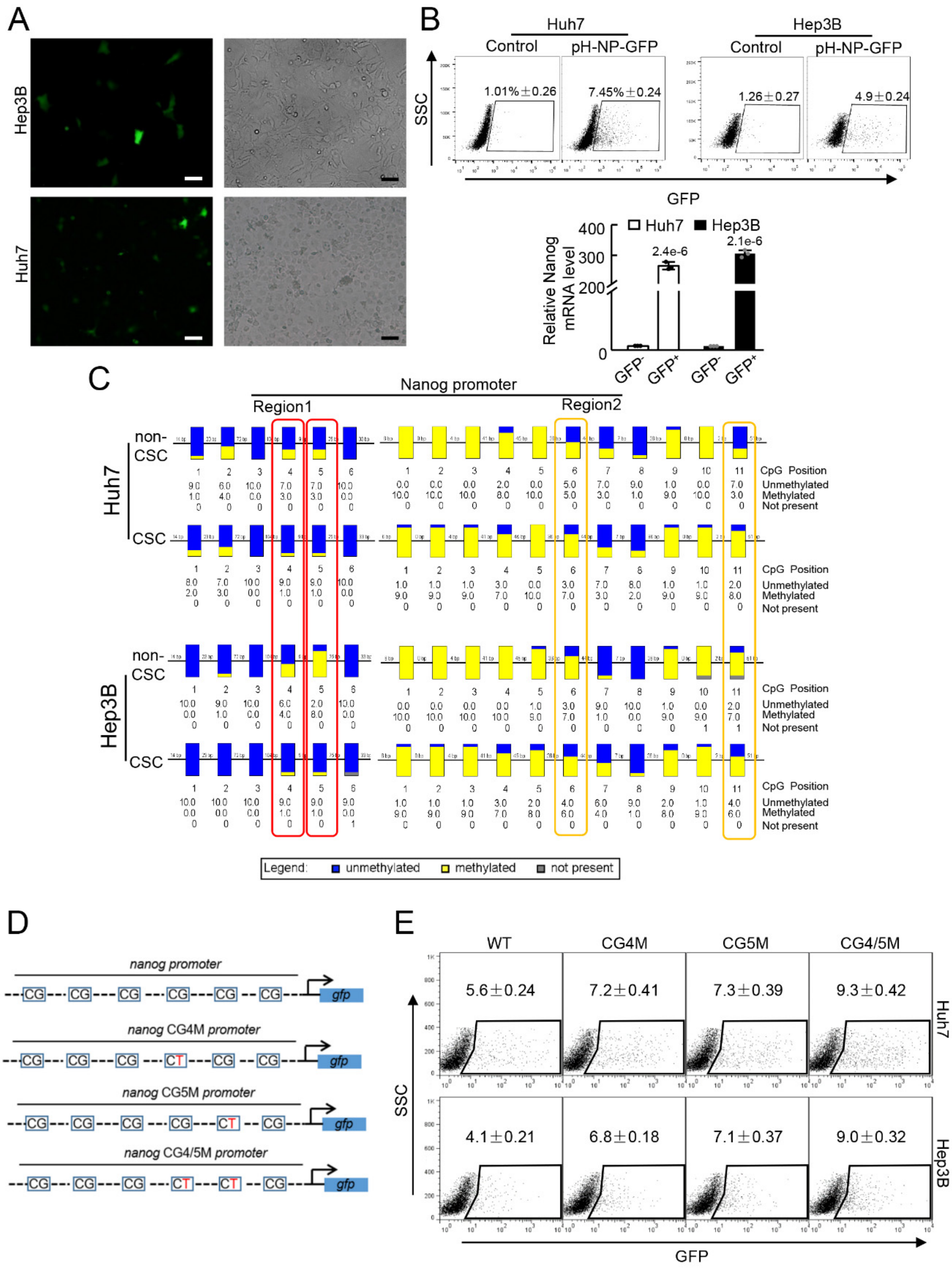

Figure S1. Methylation of the promoter regulated Nanog expression in tumor cells. A) Representative images of GFP+ cells in tumor cells transfected with pH-NP-GFP under a microscope. Scale bar, 100 μm. B) GFP+ cells in tumor cells transfected with pH-NP-GFP assessed by FACS (upper panel) and expression of Nanog in sorted GFP+ and GFP- tumor

cells assessed by qRT-PCR in triplicate (lower panel). Data are shown as the mean ± SD from triplicate experiments. P value was assessed by Student's t test. C) BSP analysis showing different methylation patterns of the *Nanog* promoter between CSCs and non-CSCs. Region1, sequence in *Nanog* promoter amplified by BSP primer Region1; Region2, sequence in *Nanog* promoter amplified by BSP primer Region2. CGs in the red square showed much lower methylation levels in the CSCs than in the non-CSCs, and the CGs in the orange square showed different methylation tendencies between the two subsets from the two cell lines. Ten clones were sequenced for each region in the nanog promoter. D), Schematic of a plasmid expressing GFP initiated by a *Nanog* promoter harboring a single nucleotide mutation at CG4 (pH-NP-GFP-CG4M), CG5 (pH-NP-GFP-CG5M) and at both CG4 and CG5 (pH-NP-GFP-CG4/5M). E) Representative images of FACS analysis of GFP^+ cells in tumor cells transfected with different GFP expression plasmids harboring the WT *Nanog* promoter or mutant promoter (CG4M, CG5M, and CG4/5M). Data are shown as the mean ± SD from at least triplicate experiments.

Figure S2

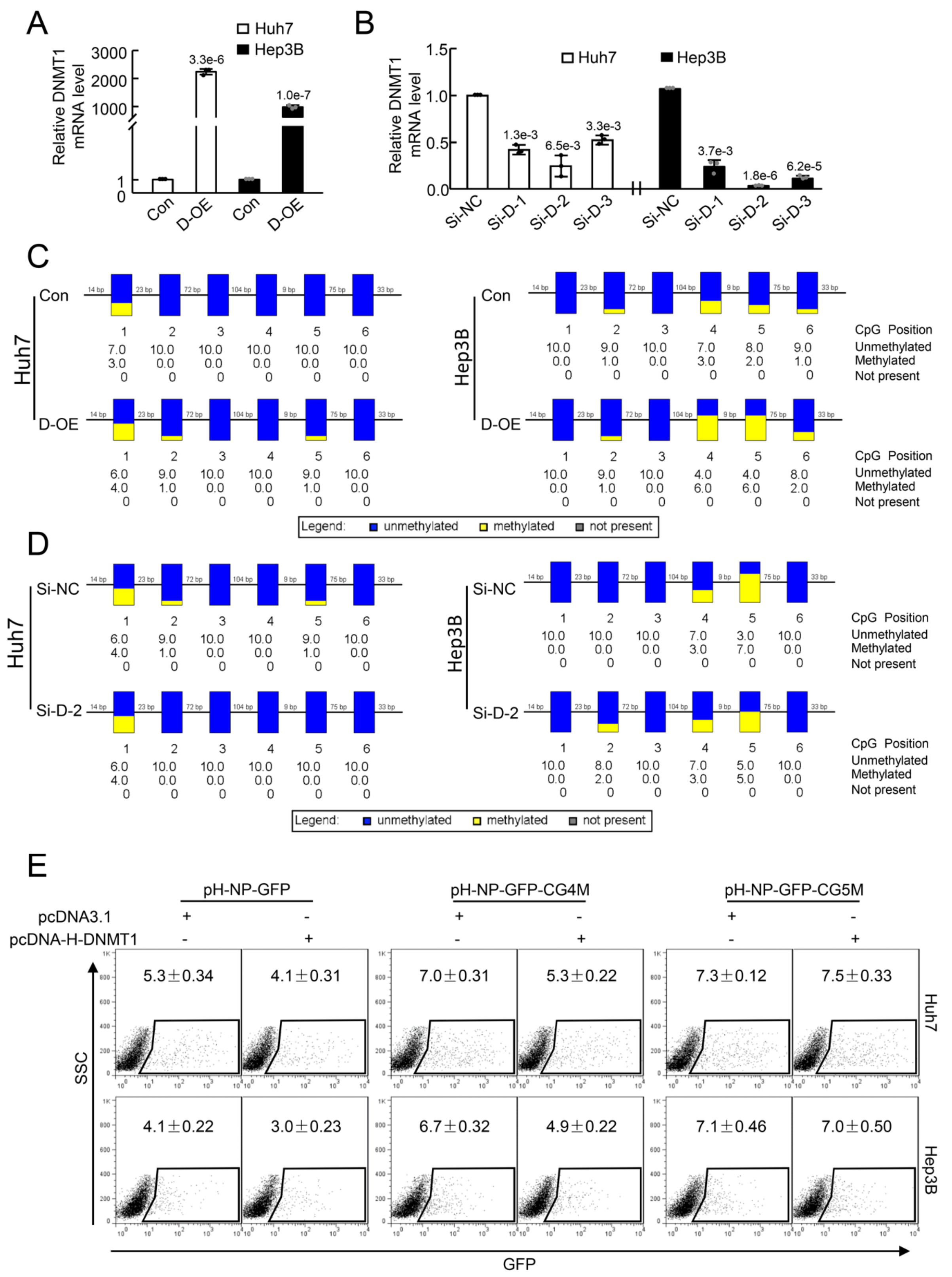

Figure S2. DNMT1 suppressed Nanog expression by methylating the *Nanog* promoter in tumor cells. A-B) Expression of DNMT1 in tumor cells transfected with a plasmid expressing DNMT1 (A) or siRNAs targeting DNMT1 (B) assessed by qRT-PCR in triplicate. Data are

shown as the mean ± SD. P value was assessed by Student's t test (A) or one-way analysis of variance (ANOVA) with Dunnett-t test in comparison with Si-NC (B). C-D) Methylation pattern of the *Nanog* promoter in tumor cells with DNMT1 upregulation (C) or downregulation (D) assessed by BSP analysis. Ten clones were sequenced for each CG in the *Nanog* promoter. E) Representative images of FACS analysis of GFP^+ cells in tumor cells cotransfected with different GFP expressing plasmids (pH-NP-GFP, pH-NP-GFP-CG4M and pH-NP-GFP-CG5M) and pcDNA-H-DNMT1 or pcDNA3.1. Data are shown as the mean ± SD from at least triplicate experiments.

Figure S3

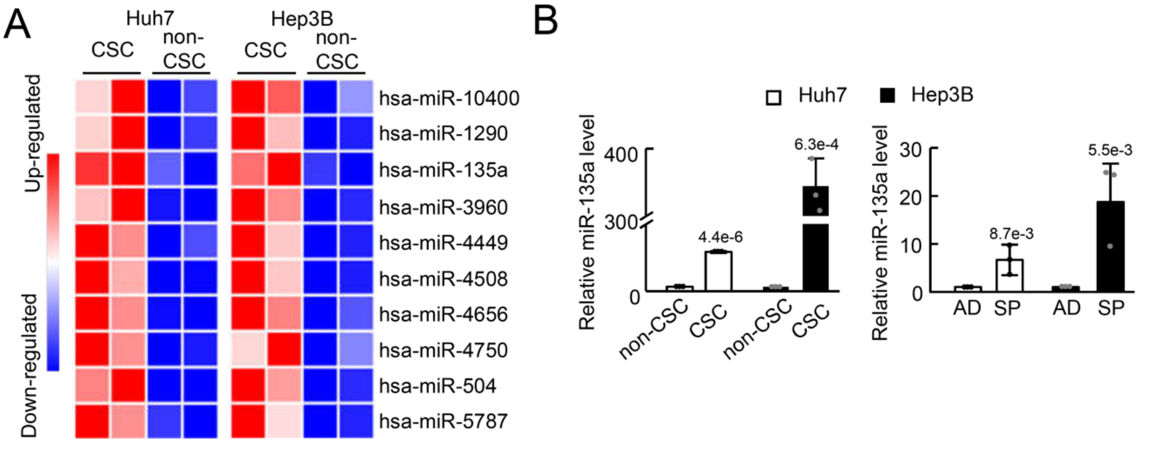

C

<table>
<tr><th>transcript name</th><th>leftmost position of predicted target site</th><th>folding energy (in -Kcal/mol)</th><th>heteroduplex</th><th>p value</th></tr>
<tr><td rowspan="3">NM_001130823.2 Homo sapiens DNA (cytosine-5-)-methyltransferase 1 (DNMT1), transcript variant 1, mRNA</td><td>268</td><td>-12.64</td><td>CCACACTGGCCGTCCCGGCCATC
| | | | | | : | | | |
AGTGTATCCTTATTTTTCGGTAT</td><td>1.98e-01</td></tr>
<tr><td>3411</td><td>-12.1</td><td>TACTGGAGCGACGAGGAGGCCGTG
| | | | | : : | : | | | : | :
AGTGTATC-CTTATTTTTCGGTAT</td><td>2.36e-01</td></tr>
<tr><td>4207</td><td>-14.3</td><td>AGAC-TAGGAG- -GCGGGCCATC
| | | | | | | :   : : | | | | |
AGTGTATCC TTATTTTTCGGTAT</td><td>2.14e-01</td></tr>
</table>

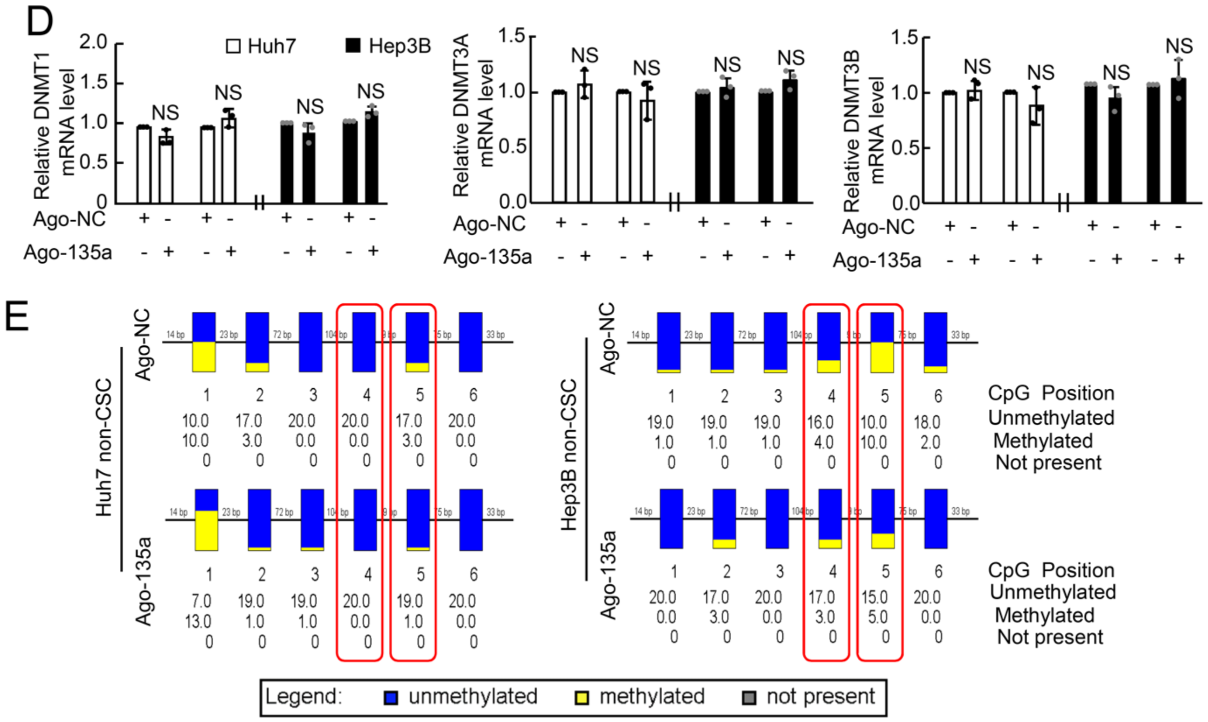

Figure S3. miR-135a regulated Nanog expression by targeting DNMT1. A) Expression of the top 10 upregulated miRNAs in CSCs and non-CSCs assessed by RNA-seq. B) Expression of

miR-135a in sorted non-CSC and CSC or spheroids (SP) from sphere formation assays and coherently cultured tumor cells (AD) assessed by qRT-PCR in triplicate. C) Schematic of miR-135a binding sites in DNMT1 mRNA predicted by RNA22 V2.0 tool. D) Expression of DNMTs mRNA in tumor cells with miR-135a upregulation (Ago-135a) or miR-135a downregulation (Ant-135a) analyzed via qRT-PCR in triplicate. E) Methylation pattern of Nanog promoter in non-CSCs with miR-135a upregulation (Ago-135a) or the control (Ago-NC) assessed by BSP analysis. Twenty clones were sequenced for regions of the nanog promoter. Data in (B) and (D) are shown as the mean ± SD. P value was assessed by Student's t test. NS, no significant difference.

Figure S4

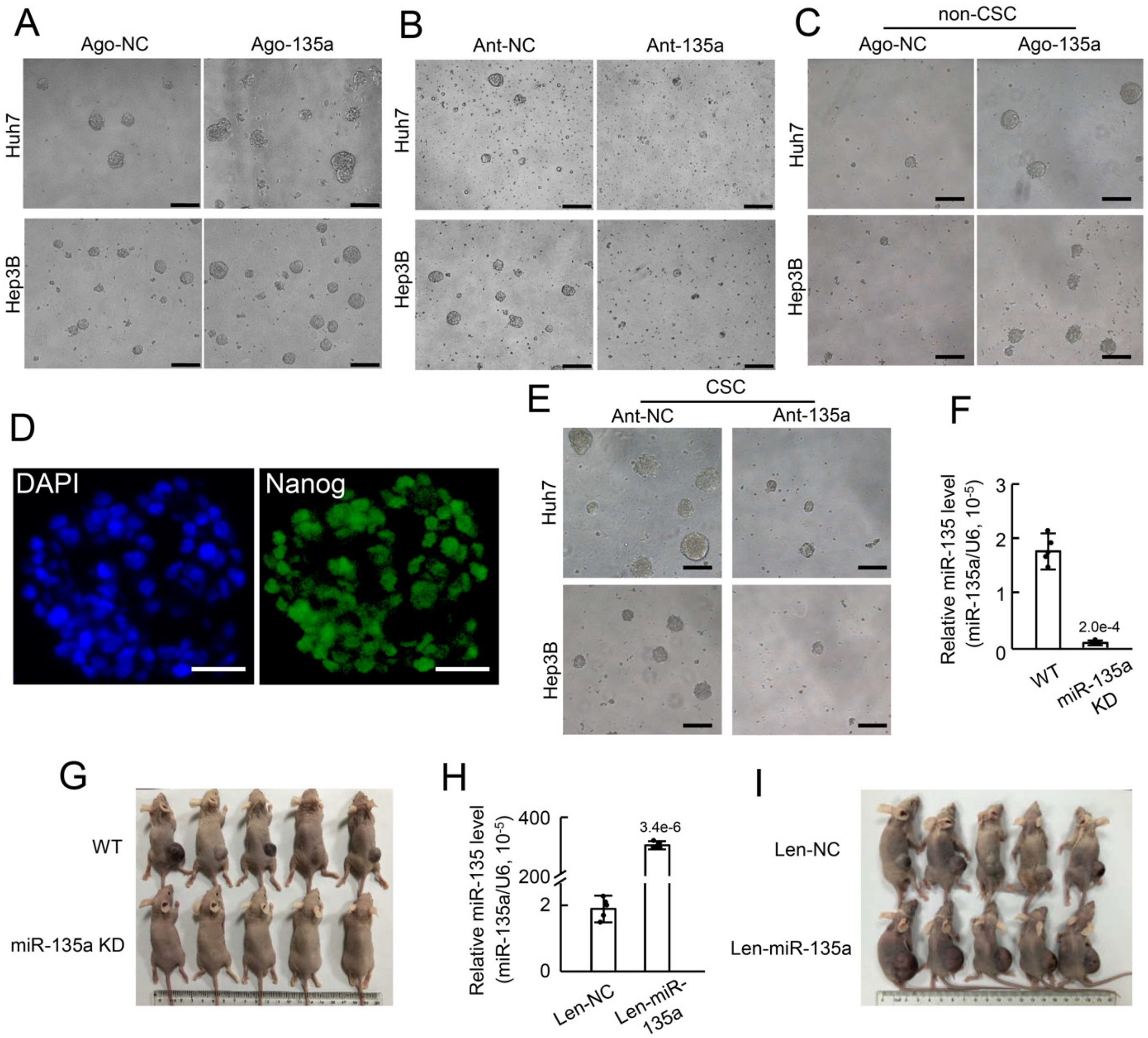

Figure S4. miR-135a enhanced the CSC capacity of tumor cells and activated Nanog expression in vitro and in vivo. A-C, E) Representative images of spheres from tumor cells with miR-135a upregulation (A) or miR-135a downregulation (B), non-CSCs with miR-135a upregulation (C) and CSCs with miR-135a downregulation (E). Scale bars, 200 µm. D) Representative images of Nanog immunostaining in spheroids derived from non-CSCs with miR-135a upregulation. Scale bars, 25 µm. F) Expression of miR-135a in tumors derived from Huh7 cells with (miR-135a KD) or without (WT) miR-135a knockdown assessed by qRT-PCR. Data are represented as the mean ± SD (n = 5 in WT and n= 4 in miR-135a KD). G) Representative images of mice with tumors derived from Huh7 cells with or without miR-135a knockdown. n=5 each group. H) Expression of miR-135a in tumors receiving Len-miR-135a or Len-NC assessed by qRT-PCR. Data are represented as the mean ± SD (n = 5 each group). I) Representative images of mice with tumors receiving Len-miR-135a or Len-NC. n=5 each group. P value in (F) and (H) was assessed by Student's t test

Figure S5

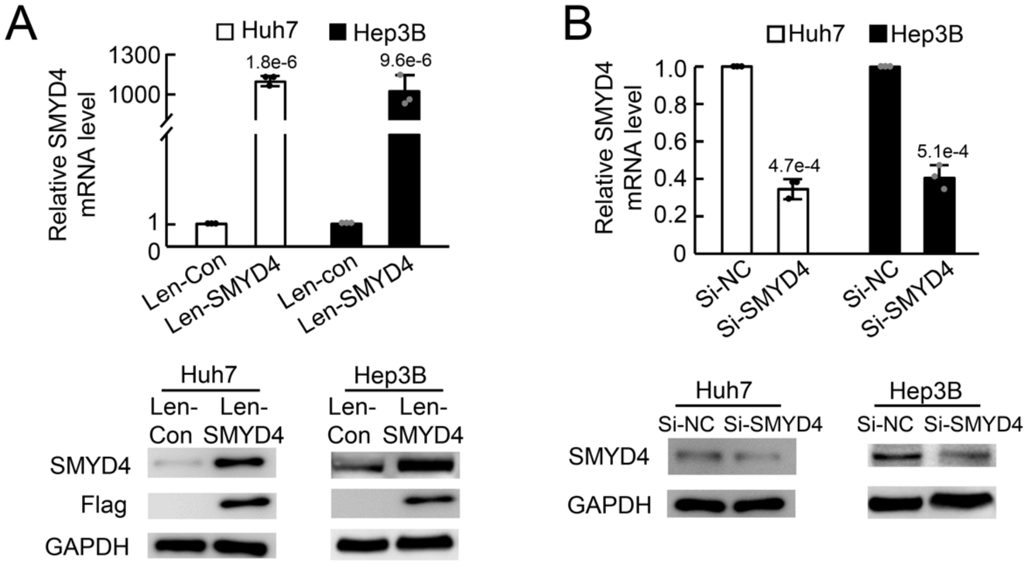

Figure S5. SMYD4 expression in tumor cells with SMYD4 modulation. A-B) Expression of SMYD4 in tumor cells transfected with lentivirus expressing SMYD4 (A) or siRNAs targeting SMYD4 (B) assessed by qRT-PCR and WB in triplicate. The image of GAPDH in (A) was also used in Figure 4C, and the image of GAPDH in (B) was also used in Figure 4D. Data are shown as the mean ± SD from triplicate experiments, P value was assessed by Student's t test.

Supplementary Tables

Table S1. Primer sequences for PCR

mRNA primers	Primer sequence	
GAPDH	Sense	5-AGAAGGCTGGGGCTCATTTG-3
	Antisence	5-AGGGGCCATCCACAGTCTTC-3
DNMT1	Sense	5-AAGAATTATCCGAGGAGGGCTA-3
	Antisence	5-GTTCAGCTCTAATCCCAGTTACTTG-3
DNMT3A	Sense	5-CAGAGGCACCGTTCACCAGAG-3
	Antisence	5-CTCCGTCCTTTCGGTCCTCC-3
DNMT3B	Sense	5-GCAACCATGTGGACGAGTCC-3
	Antisence	5-TCTCCACTGTCTGCCTCCACC-3
Nanog	Sense	5-GATGCCTCACACGGAGACTGTC-3
	Antisence	5-GCGACACTCTTCTCTGCAGAAGTG-3
SMYD4	Sense	5-CCTCTTCACTTCTTCAACCTGAGG-3
	Antisence	5-CGTCTCATACTGACCCAGGTGG-3
miRNA primers		
miR-135a	Sense	5-TATGGCTTTTTATTCCTATGTGA-3
	Antisence	5-TGCTGTCAACGATACGCTACG-3
U6	Sense	5-GCTTCGGCAGCACATATACTAAAAT-3
	Antisence	5-TGCTGTCAACGATACGCTACG-3
BSP primers		
Region1	Sense	5-GAATGAGTTAAAGAGTTTTGTTTTT-3
	Antisence	5-AACACCTACCTTAATTTCCTTTAAT-3
Region2	Sense	5-GTTTTGTTGTTTAGGTTGGAGTA-3
	Antisence	5-TTCCAACTTTTAAATCAAAAATATAAT-3
ChIP primers		
NPS1	Sense	5-GGGTTTGGGAATAGGAAGGA-3
	Antisence	5-TGGTTTCTTGTCTATCCCTCCTC-3
NPS2	Sense	5-GGCTGGTTTCAAACTCCTGACT-3
	Antisence	5-TCTAGGTTCACCACGTTTCCAAC-3
NPS3	Sense	5-ATTTGTTGCTGGGTTTGTCTTCA-3
	Antisence	5-ATCCCGTCTACCAGTCTCACCAA-3

Table S2. Antibodies for western blotting and flow cytometry.

Antibody	Manufacturer	Product number
For western blotting and immunofluorescence		
GAPDH	ABclonal Technology	AC001
DNMT1	ABclonal Technology	A17474
DNMT3A	ABclonal Technology	A2065
DNMT3B	ABclonal Technology	A7239
Nanog	Cell Signaling Technology	4903
SMYD4	ABclonal Technology	A7310
Anti-rabbit IgG (H+L)	Cell Signaling Technology	4412
Anti-rabbit IgG	Cell Signaling Technology	7074
For flow cytometry		
Nanog	eBioscience	53-5761-80
